# Expert Opinion: A Call for Early and Appropriate Symptomatic Treatment in Acute Respiratory Infections to Prevent Escalation

**DOI:** 10.3390/healthcare13212738

**Published:** 2025-10-29

**Authors:** Peter Kardos, Andrzej Fal, André Gessner, Ernest Kuchar, Christian Ude, Ludger Klimek

**Affiliations:** 1 Lung, Allergy and Sleep Centre Frankfurt at Red Cross Maingau-Hospital, 60316 Frankfurt am Main, Germany; 2 Collegium Medicum, Cardinal Stefan Wyszynski University, 01-938 Warsaw, Poland; a.fal@uksw.edu.pl; 3 Institute for Clinical Microbiology and Hygiene, University Clinic Regensburg, 93053 Regensburg, Germany; andre.gessner@klinik.uni-regensburg.de; 4 Department of Pediatrics with Clinical Assessment Unit, Medical University of Warsaw, 02-091 Warsaw, Poland; ernest.kuchar@wum.edu.pl; 5 Stern Apotheke, 64293 Darmstadt, Germany; c.ude@stern-apotheke-darmstadt.de; 6 Institute of Pharmaceutical Chemistry, Goethe University Frankfurt, 60323 Frankfurt am Main, Germany; 7 Centre for Rhinology and Allergology, 65183 Wiesbaden, Germany; ludger.klimek@allergiezentrum.org

**Keywords:** acute respiratory infection, common cold, acute cough, symptomatic treatment, phytopharmaceuticals, antimicrobial stewardship

## Abstract

Acute respiratory infections are highly prevalent and significantly impair quality of life and productivity. Despite their impact, they are often not managed according to best medical practice. A lack of knowledge about symptomatic therapies leads to mis- and under-treatment. Thus, this narrative expert opinion paper aims to highlight the importance of appropriate and early symptomatic treatment in order to assist informed therapeutic decision making and advance efforts to reduce antibiotics misuse. Clinical and mechanistic evidence supports the use of several herbal and synthetic non-antibiotic treatment options. While acute respiratory infections are usually self-limiting, treatment alleviates symptom severity, reducing the risk of inflammatory escalation. Timing of the intervention is crucial, as early initiation shortens illness duration. In conclusion, optimizing the management of acute respiratory infections could relieve the individual and societal burden of illness and slow the increase in antimicrobial resistances.

## 1. Introduction

The common cold, an umbrella term for acute upper respiratory tract infections, has substantial prevalence, peaking during the winter months in Europe [1,2]. Adults have, on average, 2–4 colds per year and children 5–8, which typically last 7–10 days. Although self-limiting, acute respiratory infections pose a significant burden on patients’ quality of life and substantially impact society due to loss of productivity, absenteeism and healthcare usage. Despite recent awareness campaigns highlighting the typically viral etiology of these diseases, irrational antibiotic treatment of uncomplicated respiratory infections remains common, contributing to the continuous and rapid increase in antimicrobial resistance [3,4]. A Europe-wide analysis revealed that almost 50% of ambulant antibiotic prescriptions were filled for respiratory infections, resulting in a reduction potential of an estimated 66–84 million packages per year, irrespective of a viral cause [3]. Driver analyses have shown that inappropriate antibiotic prescriptions for respiratory infections are partly attributable to a lack of knowledge about evidence-based symptomatic treatments, especially among the general public [5,6]. These treatments include anti-inflammatory and mucolytic herbal and synthetic pharmaceuticals, as well as topical antiseptics. Despite healthcare professionals’ (HCPs) and patients’ interest in non-antibiotic treatments, there is still uncertainty about which therapeutic options are beneficial for alleviating and shortening this usually self-limiting disease [6].

This narrative expert opinion paper therefore aims to provide an overview of clinical and mechanistic evidence for symptomatic treatments endorsed by European guidelines and supply strategies to optimize management of acute respiratory infections in practice.

## 2. Preventing Inflammatory Escalation in Respiratory Infections

**Figure 1:**
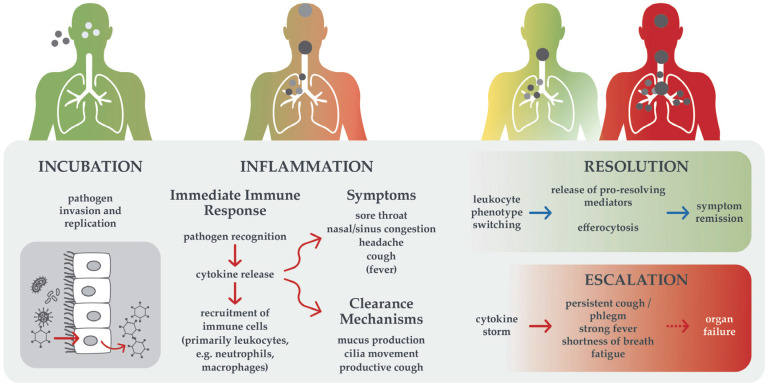
Trajectories of acute respiratory infection. Immunological processes and resulting symptoms are shown. Arrows indicate both the order of events and causal relationships. Given a functional immune system, infection with common cold pathogens usually resolves (upper trajectory). In case of immune dysregulation or dysfunction, escalation can occur (lower trajectory).

In addition to alleviating the severity and duration of symptoms, the objective of treating acute respiratory infections is to reduce the risk of inflammatory escalation. Acute respiratory infections commonly present with a symptom cascade of sore throat, nasal and sinus congestion, headaches, cough and sometimes fever [1]. Dysregulated cytokine release plays a central role in infectious diseases: these symptoms result from a uniform and excessive innate immune response, independent of the causative pathogen [2]. In the course of a respiratory infection, symptoms usually increase during the first days due to the activation of different immune processes and clearance mechanisms, as illustrated in Figure 1. Simultaneously, resolution mechanisms are triggered, leading to a gradual spontaneous decline in inflammation and, usually, remission [7].

However, in some cases, inflammation escalates due to insufficient pathogen elimination or dysregulated mediator release, leading to self-reinforcing, excessive pro-inflammatory cytokine load (“cytokine storm”) and may require intensive treatment [8]. Escalation can lead to complications, sepsis, and acute respiratory distress syndrome. While escalation is generally rare, vulnerable groups such as small children, the elderly, and people with immunodeficiency or chronic diseases are at increased risk, as recently evidenced during the SARS-CoV-2 pandemic [8,9]. Especially in chronic conditions such as asthma, bronchiectasis, and chronic obstructive pulmonary disease, acute respiratory infections comprise the main cause of exacerbations and hospitalization [2]. Preventative strategies focus on vaccination, which is unfortunately not a feasible option against most common cold pathogens and vaccination rates are usually too low to achieve herd immunity. Instead, symptomatic treatments are available to alleviate symptoms and achieve a milder clinical course.

## 3. Evidence-Based Symptomatic Treatment

Clinical and mechanistic evidence suggests that several herbal and synthetic symptomatic treatments can significantly reduce symptom severity and duration of acute respiratory infections [10,11,12,13,14]. Options endorsed in relevant European guidelines include eucalyptus extract, its main constituent 1,8-cineole, ivy, thyme, and pelargonium extracts, as well as non-steroidal anti-inflammatory drugs (NSAIDs) [15,16,17,18,19].

**Figure 2:**
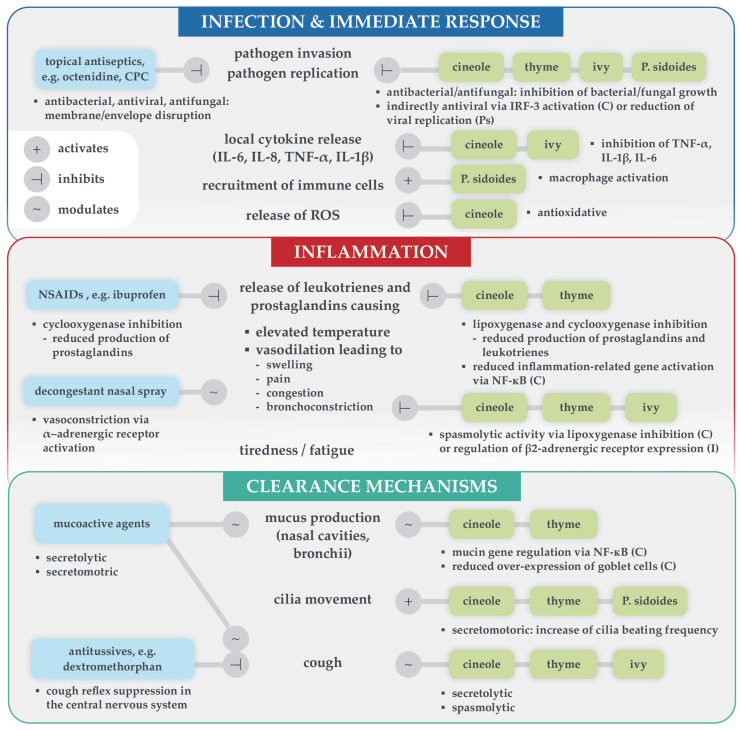
Overview of target immune processes and resulting symptoms [2] in relation to mechanisms of action of synthetic (**left**) and herbal (**right**) symptomatic treatments for acute respiratory infections. Anchors indicate activation, inhibition or modulation of the process. Detailed mechanisms are mentioned if known (octenidine [22], CPC [29], decongestant nasal spray [30], ibuprofen [31], dextromethorphan [21], cineole [24,25,27,32], ivy extract [28,33], thyme extract [34,35], *P. sidoides* extract [26]). Abbreviations: C cineole, CPC cetylpyridinium chloride, I ivy, IL interleukin, IRF interferon regulatory factor, NF-κB nuclear factor kappa B, NSAIDs non-steroidal anti-inflammatory drugs, Ps *Pelargonium sidoides*, ROS reactive oxygen species, TNF tumor necrosis factor.

Briefly, 1,8-cineole reduced bronchitis symptoms and cough significantly and with clinical relevance after 4 days in a placebo-controlled trial [12]. In addition to efficacy, timing, i.e., early intervention, is vital for accelerated symptom relief: an exploratory trial demonstrated that starting 1,8-cineole within 24 h of symptom onset shortened common-cold duration by approximately 1.5 days [10]. Ivy extract has been shown to significantly reduce cough frequency at day 7 in children versus baseline, and several studies have shown reduced cough and bronchoconstriction [13]. A fixed thyme/primrose extract reduced cough score by 47% in patients with acute bronchitis versus placebo in a double-blind RCT [11]. A Cochrane review demonstrated low-to-moderate efficacy for *Pelargonium sidoides* extract against acute rhinosinusitis and common cold [14], while another Cochrane review showed that NSAIDs can significantly relieve cold-related pain and sneezing, but not symptom score, duration or cough [20]. Additionally, topical antiseptics and herbal extracts can effectively reduce pathogen load. Treatment options targeting singular symptoms include nasal decongestants, synthetic mucoactive agents, or antitussives. However, many antitussives are not effective against acute cough [21]. Pathophysiological processes, the resulting symptoms and mechanisms of action of effective synthetic (left) and herbal (right) symptomatic treatment options are summarized in Figure 2. Mechanistic evidence is presented grouped by their target process below.

### 3.1. Pathogen Elimination

Topical antiseptics octenidine and cetylpyridinium chloride act directly by disrupting the integrity of lipid membranes, thus showing a broad unspecific antiseptic activity effective against various pathogens with low risk of resistance development [22]. Herbal treatments, on the other hand, have been shown to inhibit bacterial and fungal growth—demonstrated in preclinical studies [23]—and to act indirectly antiviral by upregulating cellular defense mechanisms [24,25,26].

### 3.2. Cytokine Regulation

Cineole and ivy extract have been shown to inhibit the release of pro-inflammatory cytokines, tumor necrosis factor α (TNF-α), and interleukins 1 and 6, thereby reducing local pro-inflammatory load [27,28].

### 3.3. Immune Cell Regulation

Immune cells, primarily leukocytes, are recruited to sites of inflammation. *Pelargonium sidoides* extract has been shown to boost this immune response by macrophage activation [26].

### 3.4. Antioxidation

Reactive oxygen species (ROS) are released upon apoptosis of infected cells, leading to the release of downstream mediators and causing harm to surrounding healthy cells. Cineole has been shown to have antioxidative effects, like many other plant extracts [27].

### 3.5. Prostaglandin Regulation

Cineole, thyme extract, and NSAIDs inhibit the production of prostaglandins via the cyclooxygenase pathway [24,31,33,34]. Prostaglandins (as well as leukotrienes) are the cause of most symptoms, as their release leads to increasing body temperature and vasodilation, which in turn results in swelling, congestion, pain and bronchoconstriction, if the lungs are affected. Nasal decongestants target α-adrenergic receptors in the nose to reduce local vasodliation and swelling [30].

### 3.6. Secrete Mobilization

Herbal medicines support intrinsic clearance mechanisms, as many have mucolytic effects and secretomotoric effects via mucin gene regulation and cilia activation [34,36,37]. There are synthetic mucoactive substances available too, e.g., erdosteine, which has mucolytic effects and activates the cilia [33].

### 3.7. Cough Relief

Spasmolytic action has been demonstrated for plant-isolated compounds such as 1,8-cineole and α-hederin (contained in ivy extract) via modulation of α- and β-adrenergic receptors, respectively, in the respiratory tract, as well as for other plant extracts [28,35,37]. Synthetic antitussives such as dextromethorphan primarily target the central nervous system [21].

## 4. Strategies to Optimize Treatment of Acute Respiratory Infections

There is a need for effective symptomatic treatment of acute respiratory infections, not least to reduce antibiotic misuse. Symptom relief and shortening of disease duration with herbal and synthetic therapeutics is both feasible and in compliance with current guideline recommendations [15,16,17,18,19]. Yet, approximately 50% of ambulant antibiotic prescriptions for respiratory infections could be avoided due to viral cause [6]. To improve management of acute respiratory infections in practice, actions on both the individual physician, pharmacist, and patient level as well as the institutional level are necessary.

While HCPs were generally well educated regarding the efficacy of antibiotics, they need to be more familiar with effective non-antibiotic treatment options, their optimal application, and how their use contributes to antimicrobial stewardship efforts [6]. The benefit of early treatment on quality of life and activity impairment should be emphasized, highlighting that the old conviction ‘treatment does not affect symptom duration’ has been disproven [10,38]. This could be achieved by common cold-specific continuing medical education programs (CME) and additional information material provided by pharmaceutical companies. Additionally, the position of symptomatic treatments in guidelines must be strengthened. Therefore, large trials with consistent and comparable endpoints would be most useful, but require additional funding and support from non-profit or government organizations to be feasible. Expert societies or international consensus conferences should work together to reduce contradictory or inconsistent recommendations, e.g., between specialist and general practitioner guidelines (cf. [15,16]).

Patients have demonstrated significantly less knowledge about the efficacy of antibiotics in the context of respiratory infections compared to HCPs [6]. They require trustworthy information—especially in the light of a multitude of non-verifiable medical information provided by social media and artificial intelligence (AI) chat bots. Educational material should include red flags requiring immediate medical attention, the risks of inappropriate antibiotics use, which symptomatic treatments are effective instead and inform that they should be initiated as early as possible for maximum benefit. Pharmacists, in particular, take a central role in counseling patients with acute respiratory infections. As they are usually the first and often only point of care, they are in the unique position to provide patients with evidence-based advice and effective over-the-counter treatment options. Recommendations by physicians have a significant impact on patient perception of the necessity of treatment and feasible options as well. Thus, addressing early and effective symptomatic treatment when patients present themselves for certification of sick leave is advisable. On the institutional level, patient information websites run by government institutions, health insurances, and medical societies should be continuously updated to comply with current guidelines and antimicrobial stewardship strategies.

## 5. Conclusions

This multidisciplinary expert opinion featuring early symptomatic as opposed to antibiotic treatment of the common cold is based on both clinical experience of the authors as well as on a search of published literature. Moreover, many specialist and general medicine guidelines support effective and safe symptomatic treatment options for acute respiratory infections. It is imperative to implement evidence-based knowledge on the optimal management of acute respiratory infections among both HCPs and patients, emphasizing the benefit of early treatment initiation and the necessity to avoid inappropriate antibiotics use.

## Data Availability

No new data were created or analyzed in this study.
